# Ternary MXene-loaded PLCL/collagen nanofibrous scaffolds that promote spontaneous osteogenic differentiation

**DOI:** 10.1186/s40580-022-00329-3

**Published:** 2022-08-27

**Authors:** Seok Hyun Lee, Sangheon Jeon, Xiaoxiao Qu, Moon Sung Kang, Jong Ho Lee, Dong-Wook Han, Suck Won Hong

**Affiliations:** 1 Department of Cogno-Mechatronics Engineering, College of Nanoscience and Nanotechnology, Pusan National University, Busan, 46241 Republic of Korea; 2 Daan Korea Corporation, Seoul, 06252 Republic of Korea; 3 BIO-IT Fusion Technology Research Institute, Pusan National University, Busan, 46241 Republic of Korea; 4 Engineering Research Center for Color-Modulated Extra-Sensory Perception Technology, Pusan National University, Busan, 46241 Republic of Korea

**Keywords:** MXene nanoparticles, Nanofibrous matrices, Electrospinning, Osteogenic differentiation, Bone tissue engineering

## Abstract

Conventional bioinert bone grafts often have led to failure in osseointegration due to low bioactivity, thus much effort has been made up to date to find alternatives. Recently, MXene nanoparticles (NPs) have shown prominent results as a rising material by possessing an osteogenic potential to facilitate the bioactivity of bone grafts or scaffolds, which can be attributed to the unique repeating atomic structure of two carbon layers existing between three titanium layers. In this study, we produced MXene NPs-integrated the ternary nanofibrous matrices of poly(L-lactide-*co*-*ε*-caprolactone, PLCL) and collagen (Col) decorated with MXene NPs (i.e., PLCL/Col/MXene), as novel scaffolds for bone tissue engineering, via electrospinning to explore the potential benefits for the spontaneous osteogenic differentiation of MC3T3-E1 preosteoblasts. The cultured cells on the physicochemical properties of the nanofibrous PLCL/Col/MXene-based materials revealed favorable interactions with the supportive matrices, highly suitable for the growth and survival of preosteoblasts. Furthermore, the combinatorial ternary material system of the PLCL/Col/MXene nanofibers obviously promoted spontaneous osteodifferentiation with positive cellular responses by providing effective microenvironments for osteogenesis. Therefore, our results suggest that the unprecedented biofunctional advantages of the MXene-integrated PLCL/Col nanofibrous matrices can be expanded to a wide range of strategies for the development of effective scaffolds in bone tissue regeneration.

**Graphical Abstract:**

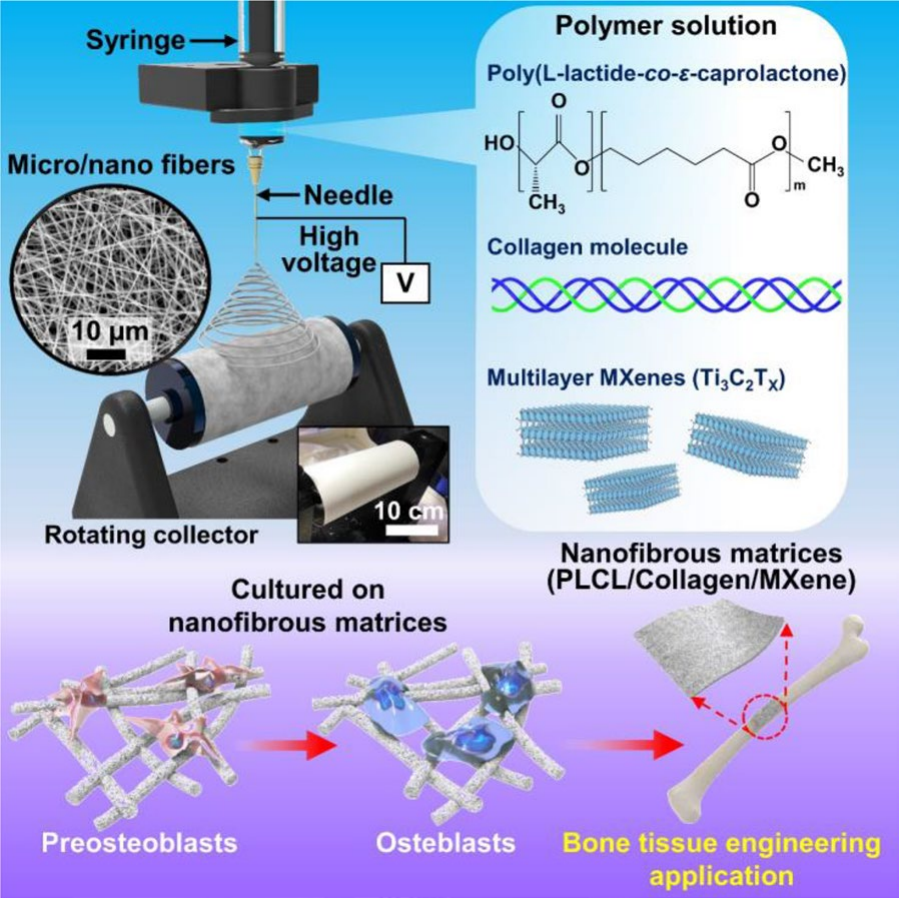

**Supplementary Information:**

The online version contains supplementary material available at 10.1186/s40580-022-00329-3.

## Introduction

The novel bone graft materials have developed orthopedic and dental therapy that allow the reconstruction of irreversibly damaged bone tissues. Traditional orthopedic and dental implants have employed bioinert metals and ceramics, including stainless steel, titanium alloys, zirconium, and alumina [1, 2]. Bioinert bone grafts have good mechanical strength as well as corrosion and crack resistance, low weight, and suitable biocompatibility, but often fail osseointegration due to low bioactivity [3, 4]. To overcome this issue, the tissue engineering approach has adopted a completely different approach by using scaffolds of different structures and materials, compared to those conventional bone grafts. The former contains sponge-like bone tissue analogs, nanofibrous matrices, hydrogel, and surface nanotopography, which are cytocompatible and beneficial for combining with surrounding tissues [5, 6]. Meanwhile, the latter focuses on the bioactive materials that can promote the behaviors of surrounding cells, such as adhesion, migration, proliferation, and differentiation [7].

A number of recent studies proposed the excellent potentials of two-dimensional (2D) nanomaterials that support or promote various types of cell growth and tissue regeneration [8, 9]. The materials system includes transition metal dichalcogenide, metal–organic framework, and graphene derivatives, suggesting the nanocomposite of the 2D nanomaterials with other biomaterials, such as ceramics and polymers. Among these nanomaterials, MXene, 2D transition metal carbides and carbonitrides, have represented several attractive properties for biomedical applications, such as tissue engineering scaffolds, therapeutics, and biosensors [10, 11]. In particular, its exceptional osteogenic activity and cytocompatibility could lead to enhanced osseointegration, osteoconduction, and osteogenesis after bone graft transplantation [12–14]. On the other hand, the electrospun fibers as an extracellular matrix (ECM) are one of the most usefully developed scaffolds for bone tissue regeneration due to their ease of access in morphological and chemical modification with cost-effective and scale-up manufacturing processes [15]. Moreover, the electrospun nanofibrous matrices can provide cells with the biomimetic microenvironment similar to the native ECMs [16].

In the past few decades, considerable research on viable strategies for bone tissue scaffolds has been widely developed to accelerate the bone formation and reconstruction in an effective manner [17–19]. However, complete bone regeneration in an artificial environment remains an unresolved challenge in finding the ideal ECM but provides sufficient information for tissue engineering studies [20]. For example, previous works on nanofibrous matrices as bone tissue engineering (BTE) scaffolds have investigated various types of biocompatible or biodegradable polymers, including chitosan [21], poly(lactic-*co*-glycolic acid, PLGA) [22], poly(l-lactide, PLLA) [23], polycaprolactone (PCL) [24], poly(l-lactide-*co*-*ε*-caprolactone, PLCL) [25], etc. Recently, in some other cases, a new material system incorporated with 2D nanomaterials has emerged as a promising field of research and has given numerous opportunities for unrevealed tissue engineering areas [26–28]. In this context, we might acknowledge that the composite forms of ECM platforms such as newly introduced 2D nanomaterials with conventional polymeric materials can be one of the solutions in BTE.

Here, we developed a simple but robust strategy in the use of MXene NPs-integrated PLCL/Col for BTE application. The PLCL/Col-based polymeric nanostructured matrix decorated with MXene nanomaterial can be an intriguing combination as a potential biomaterial with an enhanced specific biological affinity, supported by the biodegradability of the major components in the provided bone regenerative ECM. To our best knowledge, an experimental approach for the direct incorporation of PLCL/Col matrices with MXene NPs is extremely rare although PLCL has been widely utilized as a scaffold in advanced tissue engineering mainly due to its exceptional tissue compatibility, rubber-like elasticity, and suitable degradability [29–31]. The main drawback in the use of the PLCL has lied in lack of affinity to the cells, but some other intermediating biomolecules, such as collagen (Col), fibronectin, or RGD peptides, have been utilized to efficiently help a cell adhesion to the prepared matrices [32, 33]. Therefore, by adding the monomeric form of type I Col in the matrix [34], we developed a MXene-decorated PLCL/Col nanostructured material as a novel scaffold toward BTE. We postulate that this newly designed material induces the two members of the integrin family (i.e., α_1_β_1_ and α_2_β_1_ integrins) that are expressed from the cell membrane, which is highly beneficial for the cell affinity. The characteristic features of the prepared bio-interfacial materials were crucially defined to explore the unrevealed cell responses on our scheme, and the related affirmative interactions of MC3T3-E1 preosteoblasts were fully evaluated with a series of cellular behaviors, such as attachment, proliferation, and osteogenic differentiation.

## Experimental

### Preparation and characterizations of MXene NPs

Ti_3_AlC_2_ powder (≥ 98.0%, 200 mesh) was purchased from 11 Technology Co., Ltd. (Jilin, China). The layered MXene Ti_3_C_2_T_x_ nanosheets were synthesized by etching Ti_3_AlC_2_ powder with the HF solution. Briefly, 2 g of Ti_3_AlC_2_ powder is slowly added into 50% (w/v) HF (20 ml) and the solution is stirred for 48 h at 50 °C in an oil bath. The multilayer Ti_3_C_2_T_x_ is obtained by washing with DI water via centrifugation at 3500 rpm for 3 min several times until the pH of the supernatant reached ~ 6. The as-prepared dry multilayer Ti_3_C_2_T_x_ (2 g) was re-dispersed into DI water (50 ml), followed by the addition of DMSO (40 ml) by stirring for 24 h. Finally, after the vacuum filtration and drying process, the exfoliated Ti_3_C_2_T_x_ NPs were obtained as a membrane form, which was weighted to prepare the concentrated solution dispersed in DI water. In our experimental condition, the yield was found to be more than 80% from the precursor.

### Fabrication of PLCL/Col/MXene nanofibrous matrices

The PLCL/Col/MXene nanofibrous matrices were fabricated by conventional electrospinning process. Briefly, PLCL (75:25, molecular weight 40–80 kDa, BMG Inc., Kyoto, Japan) and Col (Darim Tissen, Seoul, Korea) were dissolved in 1, 1, 1, 3, 3, 3-hexafluoroisopropanol (HFIP, Sigma-Aldrich Co., St Louis, MO). PLCL and Col were contained at the concentrations of 5 and 0.5% (w/v) in the working solution of HFIP, respectively. The MXene NPs solution in DI water was prepared by sonicating for 1 h to distribute them evenly throughout the solution and was mixed with the PLCL/Col solution at the final concentration of 400 µg ml^‒1^. The mixture (10 ml) of PLCL, Col, and MXene NPs were then loaded into a syringe (Henke-Sass, Wolf GmbH, Tuttlingen, Germany) with a spinneret needle (0.5 mm). A voltage of 16 kV was applied using a DC high voltage power supply (NanoNC, Seoul, Korea). The working distance between the needle tip and the collector was 9 cm and the flow rate was 0.2 ml h^‒1^ (a summary of the experimental conditions can be found in Additional file 1: Table S1). The fabricated PLCL/Col/MXene nanofibrous matrices were collected on a steel rotating wheel covered with A4 paper. After the spinning process, the PLCL/Col/MXene nanofibrous matrices were dried overnight under vacuum at room temperature to completely remove the residual solvent. Subsequently, the fabricated nanofibrous matrices were cut into a disc shape with 9 mm diameter and then sterilized by ultraviolet light irradiation overnight prior to use.

### Physicochemical characterizations of MXene NPs and PLCL/Col/MXene nanofibrous matrices

The surface morphology of the prepared MXene NPs and all the nanofibrous matrices was observed by field emission (FE)-SEM (Carl Zeiss Supra 40VP, Oberkochen, Germany) at an accelerating voltage of 15 kV. Their crystallinity and elemental mapping were analyzed by scanning TEM (STEM) with an EDS operated at 200 kV (STEM-EDS, Talos F200X, ThermoFisher Scientific, Hillsboro, OR). The topography of all the matrices was characterized by AFM (NX10, Park Systems Co., Suwon, Korea) in air at RT. AFM imaging was performed in non-contact mode with a multi silicon scanning probe at a resonant frequency of ~ 300 kHz and image analysis was performed using XEI software (Park Systems Co.). The water contact angles of all the matrices were measured by sessile drop method using a contact angle measurement system (SmartDrop, Femtofab Co. Ltd., Seongnam, Korea). A 1 μl sessile drop of distilled water was formed on all the matrices. Compositional analysis of the PLCL/Col/MXene nanofibrous matrices was performed by FT-IR spectroscopy (Spectrum GX, PerkinElmer Inc., Waltham, MA) and XPS (AXIS Supra, Kratos Analytical Ltd., Manchester, UK). FT-IR spectra were recorded in absorption mode in the wavelength range of 400–3500 cm^−1^ with a resolution of 4.0 cm^−1^ and 16-times scanning. XPS spectra were adopted to confirm the O 1 s, N 1 s, C 1 s, Ti 2 s, and Ti 2p states.

### Cytotoxicity of MXene NPs, cell attachment and proliferation assays

A murine preosteoblastic cell line (MC3T3-E1 preosteoblasts from C57BL/6 mouse calvaria) was purchased from the American Type Culture Collection (CRL-2593™, ATCC, Rockville, MD). MC3T3-E1 cells were routinely cultured in α-Minimun Essential Medium (basal medium) supplemented with 10% (v/v) fetal bovine serum and a 1% (v/v) antibiotic antimycotic solution (including 10,000 U penicillin, 10 mg streptomycin, and 25 µg amphotericin B per ml) (all from Sigma-Aldrich Co.) at 37 ℃ in a humidified atmosphere containing 5% CO_2_. MC3T3-E1 cells are an established cell line that has been used to examine osteogenesis and bone differentiation [36]. The cytotoxicity profiles of MXene NPs were determined by CCK-8 (Dojindo Molecular Technologies Inc., Kumamoto, Japan) and LDH (Lactate dehydrogenase) assays (Takara Bio Inc., Shiga, Japan) according to the manufacturer’s instructions. A CCK-8 assay involves the quantitative measurement of DH enzyme (i.e., metabolic) activity in cells, while an LDH assay indicates membrane integrity according to the amount of LDH release [37, 38]. Briefly, the cells were seeded at a density of 5 × 10^4^ cells cells/ml in a 96-well plate and incubated for 24 h. Subsequently, the cells were treated with the increasing concentrations (0–250 µg ml^‒1^) of MXene NPs suspended in culture medium and then incubated with a CCK-8 solution for the last 2 h of the culture period (24 h and 48 h) at 37 ℃ in the dark. The absorbance was measured at 450 nm using a microplate reader (Varioskan LUX, ThermoFisher Scientific). For an LDH assay, after 24 and 48 h of incubation with the increasing concentrations (0–250 µg ml^‒1^) of MXene NPs, the supernatant from the treated cells was transferred to a new 96-well plate. Afterwards, an LDH solution was added to each well and then incubated for 30 min at RT in the dark. The absorbance was measured at 490 nm using a microplate reader. The initial attachment and proliferation of preosteoblasts on the prepared nanofibrous matrices were evaluated by a CCK-8 assay. The cells with a density of 3 × 10^4^ cells/matrix were seeded on each matrix. Following the same protocol with cytotoxicity determination, the initial attachment (4 h) and proliferation (1, 3, and 7 days) were measured.

### ALP activity assay

The early-stage marker of osteogenic differentiation was measured using an ALP assay (Abcam, Cambridge, MA). Similar to the proliferation assay, MC3T3-E1 preosteoblasts were seeded on PLCL, PLCL/Col, PLCL/MXene, and PLCL/Col/MXene nanofibrous matrices at a density of 1.0 × 10^4^ cells/matrix and then incubated in basal media for up to 14 days. ALP activity was determined by measuring the transformation of ρ-nitrophenyl-phosphate (ρNPP) to ρ-nitrophenol (ρNP) in yellow color, which is produced in the presence of ALP [39]. At the end of pre-determined incubation period, the cells were washed twice with Dulbecco’s phosphate-buffered saline (DPBS, Sigma-Aldrich Co.) and incubated in a 0.1% Triton X-100 solution (Sigma-Aldrich Co.) in Tris-buffer (10 mM, pH 7.5, Sigma-Aldrich Co.) for 10 min. Subsequently, a freshly prepared ρNPP solution (50 μl) was added to cell lysate (80 μl) of each matrix, followed by incubation for 1 h in a CO_2_ incubator. After incubation, the reaction was finished by adding a stop solution (20 μl). The absorbance was measured at 405 nm using a microplate reader and the ALP activity was calculated from the ρNP formation (μmol) divided by the volume (ml) and reaction time (min).

### Von Kossa staining

Von Kossa staining is widely used to monitor the mineralized bone nodules of the differentiated osteoblasts [36, 40]. Von Kossa is not specific for calcium ion, but positive for carbonate or phosphate ions in calcium deposits by staining them in a brownish-blackish color. The MC3T3-E1 preosteoblasts with a density of 5.0 × 10^3^ cells/matrix were seeded on PLCL, PLCL/Col, PLCL/MXene, and PLCL/Col/MXene nanofibrous matrices and cultivated in basal media under a 5% CO_2_ atmosphere at 37℃. At the end of incubation period for 1 to 21 days, the cells then were washed twice with DPBS and then fixed with 4% formaldehyde for 10 min at RT. After fixation, the cells were stained with a freshly prepared 5% silver nitrate solution for 30 min in ultraviolet light and washed thrice with DI water. Afterwards, the cells were reacted with 2% sodium thiosulfate for 5 min to remove any unreacted silver nitrate. Finally, the cells were washed thrice with DI water, air-dried and photographed using a digital camera (Olympus Optical Co., Osaka, Japan). The images were analyzed using ImageJ software (National Institutes of Health, Bethesda, MD).

### Statistical analysis

All variables were tested in three independent cultures for each experiment, which was repeated twice (n = 6). All experimental results are presented as the mean ± standard deviation (SD). The data were tested for the homogeneity of the variances using the Levene test, prior to statistical analysis. Statistical comparisons were performed using a one-way analysis of variance, followed by a Bonferroni test for multiple comparisons. Values of p < 0.05 and p < 0.01 were considered statistically significant.

## Results and discussion

### Composite preparation: MXene-integrated nanofibrous matrices

**Fig. 1:**
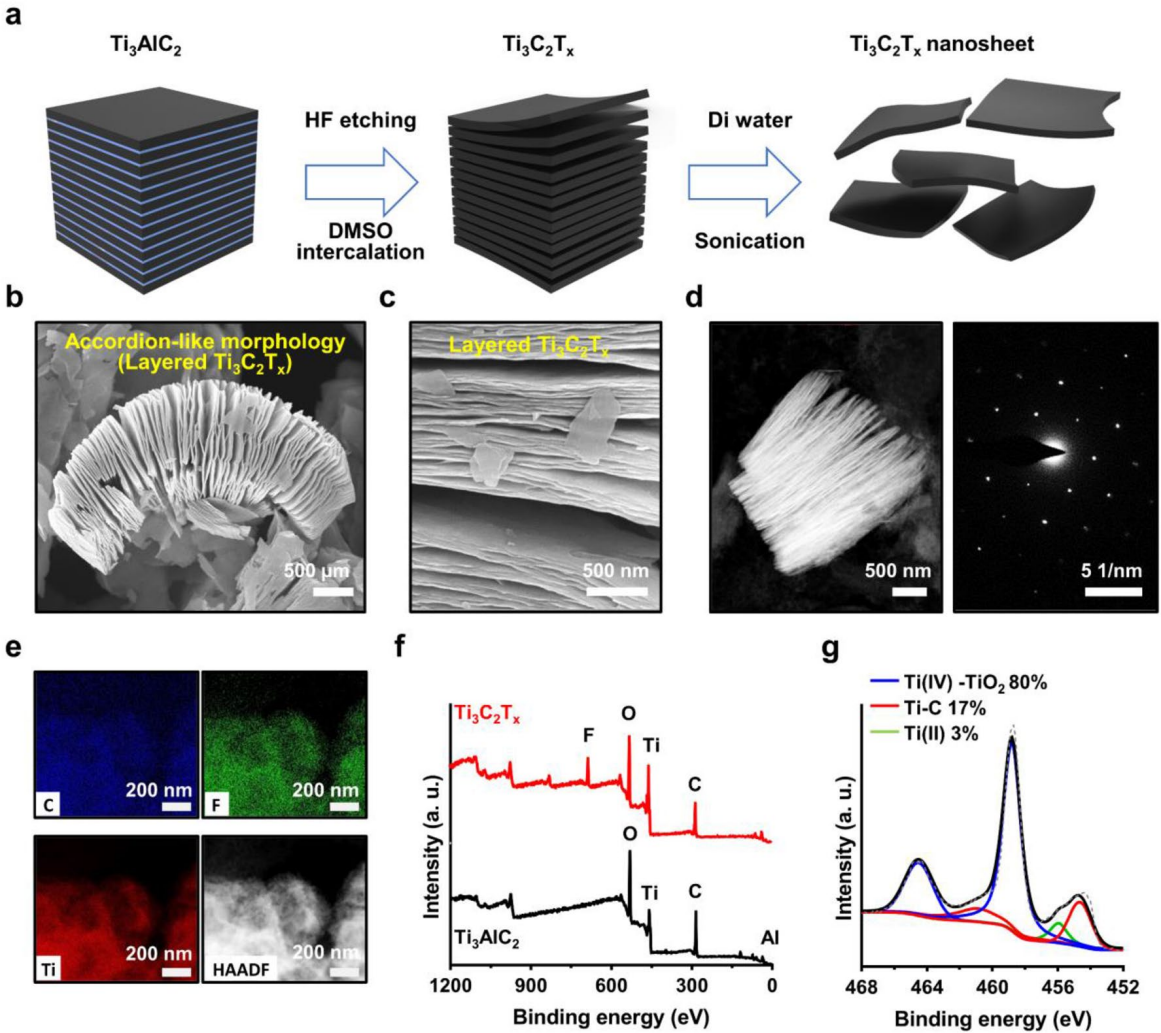
Synthesis and characterization of Ti_3_C_2_T_x_. **a** Schematic illustration of the synthesis of Ti_3_C_2_T_x_ by selective etching of the Al layer. **b** SEM image of layered Ti_3_C_2_T_x_ with typical accordion-like morphology. **c** Side view of a highly magnified SEM image in multilayer MXene. **d** HAADF-STEM image of Ti_3_C_2_T_x_ and the corresponding SEAD pattern. **e** EDS mapping profiles of Ti_3_C_2_T_x_ with C (blue), F (green), Ti (red). **f** The survey spectrum and **g** chemical component survey by XPS spectra (Ti 2p)

Optimized condition for producing 2D MXenes colloidal solution includes exfoliation of the starting material (e.g., Ti_3_AlC_2_) via an acidic treatment on the layered crystalline MAX phase, M_n+1_AX_n_ (n = 1, 2, or 3); M, A, and X are denoted by a transition metal, a layer of IIIV or IVA component, and carbon or nitrogen, respectively [41, 42]. Figure 1a schematically illustrates the scheme of the exfoliating method to prepare the MXene NPs. As presented, the first step involved the chemical intercalation of the Ti_3_AlC_2_ by a selective etching A component with hydrofluoric acid (HF). In the Al removal process from the MAX phase, a high rate of fluoride ions (F^−^) were actively interacted in the source materials, yielding the M_n+1_X_n_T_x_ (i.e., Ti_3_C_2_T_x_ phase). Here, T_x_ refers to a functional group (–F, –O, –OH), generated during the synthesis process. Because M–X bonds are chemically more stable than the M–A bonds, the selective etching of the M–A bond enabled the formation of MXenes, as described previously [43]. Next, the multilayer MXenes (i.e., layered Ti_3_C_2_T_x_) were readily expanded by the common intercalation using large organic molecules of dimethyl sulfoxide (DMSO), which weakens the binding forces between the layers, increasing the interlayer distance. Finally, the subsequent separation of layers of MXenes was yielded upon ultrasonication. Although this process is firmly established, it is important to completely remove the A layer component from MAX phase for a uniform distribution of the functional groups after the synthesis. The characteristic features of the scanning electron microscopy (SEM) image display the architectural structures of the intercalated Ti_3_C_2_T_x_ layers in nanoscale as shown in Fig. 1b, which shows an accordion-like morphology with a spatially separated configuration of the flakes, representing cross-sectional weak bonds of the multilayer MXenes for the successful exfoliation from the Ti_3_AlC_2_. A highly magnified SEM image defines a thickness range of the MXene NPs (~ 10–25 nm), consisting of individually separated layers (Fig. 1c and Additional file 1: Fig. S1). For more information, we measured the samples with high-resolution transmission electron microscopy (HRTEM) (Additional file 1: Fig. S2) and collected selected area electron diffraction (SAED) patterns as presented in Fig. 1d; the data set indicate that the MXene NP-formation, including Ti_3_C_2_ and TiO_2_, exhibits stacked multilayered-sheet features with a highly crystalline structure. Additional elemental mapping analysis by energy dispersive spectroscopy (EDS) was imaged and represented evenly distributed Ti, C, and F elements on the multilayered MXenes NPs (Fig. 1e). As shown in Figs. 1f and g, the chemical composition and the surface state were analyzed by X-ray photoelectron spectroscopy (XPS) measurement. The survey spectrum for the prepared Ti_3_C_2_T_x_ reveals the presence of Ti, F, O, and C, compared to the source MAX phase (Fig. 1f), and the high-resolution spectra (Ti 2p) on the MXene exhibited three principal peaks corresponding to Ti-C (~ 454.3 eV) and TiO_2_ (~ 458.7 eV) components, respectively (Fig. 1g). As previously reported [44, 45], the stock colloidal solution of MXene NPs was continuously oxidized in water solvent by the active reaction at the edges, leading to the formation of anatase structured TiO_2_ [46]. By this XPS measurement after the sample preparation step, we confirmed that the exfoliation of MXenes is highly reproducible to successfully transform the MAX phase into well-dispersed colloidal NPs in an aqueous solution with high stability.

**Fig. 2:**
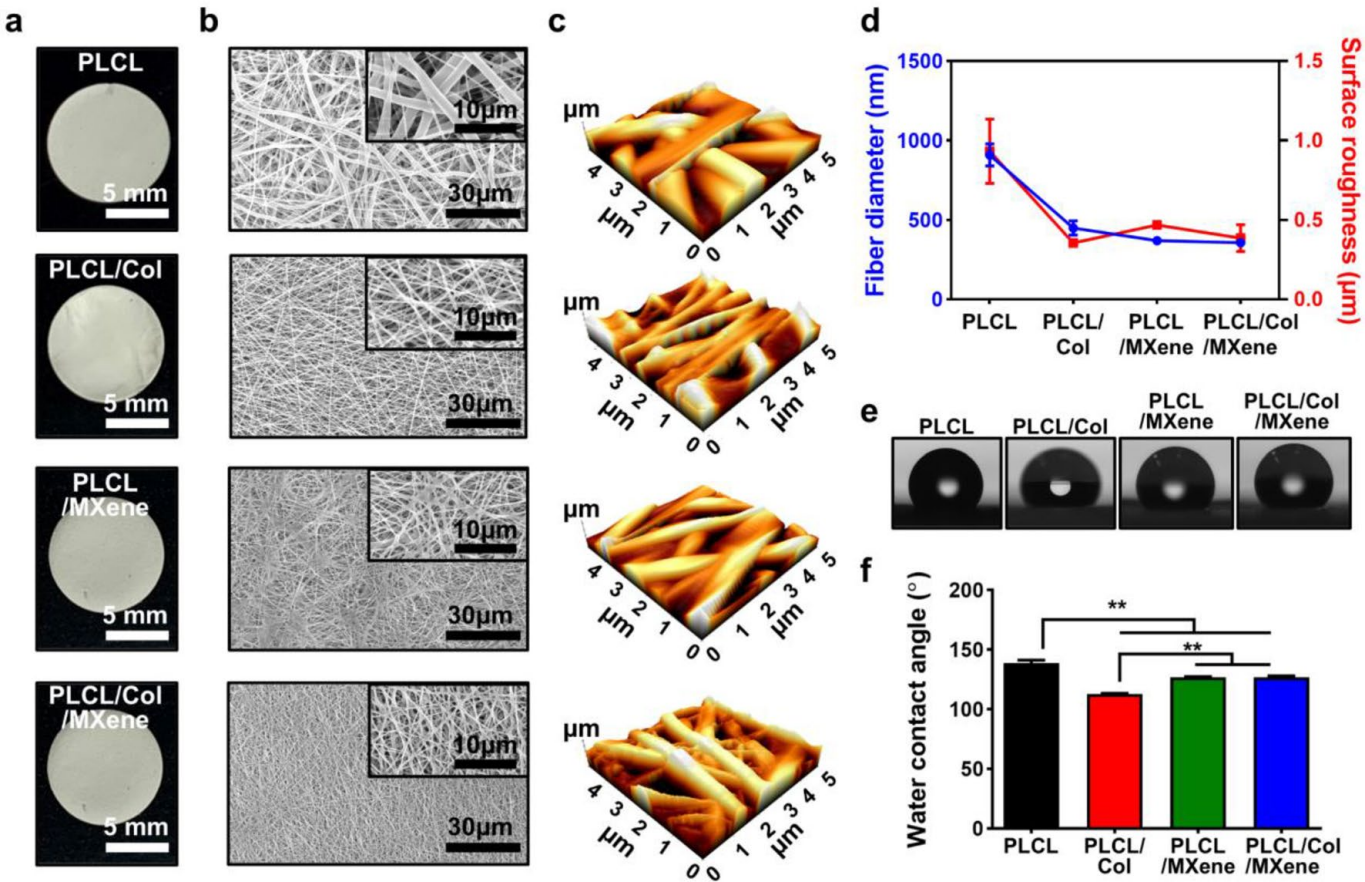
Physical properties of PLCL, PLCL/Col, PLCL/MXene, and PLCL/Col/MXene nanofibrous matrices. **a** Digital photographs of the disc-shaped nanofibrous matrices. **b** Morphological characteristics of nanofibrous matrices observed by SEM. **c** Surface topographic images and their corresponding roughness measured by AFM. **d** Diameter and surface roughness of electrospun nanofibrous matrices according to the material composition. **e**, **f** Digital photographs of the sessile droplets on each matrix with the corresponding contact angles. The data are expressed as the mean ± SD (n = 6). Asterisks (**) denote a significant difference compared to the control, **p < 0.01. All photographs and micrographs are representative of six samples with similar results

To utilize the PLCL matrix with MXene NPs, nanofibrous ECM matrices were prepared by the typical electrospinning process (see Materials and Method for details). In our experimental scheme, the combinatorial material system was set with PLCL, PLCL/Col, PLCL/MXene, and PLCL/Col/MXene to explore the unclassified cell affinity on different compositions. Thus, at the initial step, the physicochemical properties were characterized as follows. Figure 2a presents digital photographs of the produced nanofibrous matrices that have macroscopically uniform and smooth surfaces with the same appearance. When the magnified surface morphology was observed by SEM (Fig. 2b), the random networked nanofibers appeared. The morphological features were resembled the natural ECM and fully interconnected in a porous structure with a relatively uniform diameter. Magnified images within the same magnitude scale displayed interesting size changes in fiber diameter of the nanofibers. As measured, the averaged fiber diameters for each sample of PLCL, PLCL/Col, PLCL/MXene, and PLCL/Col/MXene were 908 ± 68, 449 ± 44, 368 ± 22, and 357 ± 3 nm, respectively (Fig. 2d). When other constituents such as MXene, Col, or both were mixed into the main PLCL matrix, the fiber diameters decreased by ~ 40% from the original scale of the PLCL fibers. This result can be attributed to the subtle intramolecular interactions dispersed in a common solvent (i.e., DI water), associated with a concentration of polymer solution because the viscous flow of the electrospinning solution was a controllable factor in determining the diameter of the electrospun nanofibers and morphologies [47, 48]. This phenomenon is a well-known factor to be tailored by adjusting the polymer concentration of the solution or adding other materials. In our case, by adding the MXene NPs to the polymer solution, the viscosity decreases at the same concentration condition, compared to other polymer solutions. Moreover, the mass ratio of MXene NPs in the nanofibrous matrices was carefully selected and used for the electrospinning process, taking into account the volume of the polymer solution and the results of the nanofibrous sheet (i.e., disc shape with 9 mm diameter). Based on the previous reports [13, 35], we assumed that the mass ratio of the MXene NPs in the defined area was within the range of the optimized mass ratio evaluated by a series of the experiments, such as cytotoxicity and ALP activity tests. With a similar but slightly different from the previous approach, we also postulated that the MXene NPs in the nanofiber matrices may act differently on the cell behaviors because they are embedded in the surface of the matrices rather than placed on the cell culture plates.

The surface topography and roughness of the nanofibrous matrices were also explored by using atomic force microscopy (AFM) as shown in Fig. 2c. The measured surface roughness (*Ra*) of PLCL, PLCL/Col, PLCL/MXene, and PLCL/Col/MXene nanofibrous matrices were 0.932 ± 0.202, 0.354 ± 0.021, 0.468 ± 0.003, and 0.385 ± 0.083 μm, respectively (Fig. 2d). As reported previously, the surface roughness of the fibrous matrices decreases as the individual fiber’s diameter of decreases [49, 50]. Subsequently, the surface hydrophilicity of each substrate (i.e., nanofibrous matrix) was evaluated by the water contact angle measurement using a sessile droplet (Fig. 2e, f). PLCL/Col, PLCL/MXene, and PLCL/Col/MXene nanofibrous matrices showed significantly (p < 0.01) decreased contact angles, compared to the neat PLCL samples. The nanofibrous features can mimic the native ECM topography, which is greatly advantageous due to tissue compatibility with similar structure formation composed of collagen, laminin, other fibrils, and proteoglycans that are hierarchically arranged in the nanometer range [51]. For example, the nanotopography endows stem cells and preosteoblasts with native cell niches to guide osteogenic differentiation [52], and the nanotopographic enveloping of the cell membrane spontaneously leads to integrin rearrangement. This modified intramembrane tension induces rearrangement of the cytoskeleton to influence the cell behaviors including adhesion, migration, proliferation, and differentiation [53]. Therefore, this result implies that Col and MXene NPs can be superior for cell adhesion and growth due to better hydrophilicity [54, 55].

**Fig. 3:**
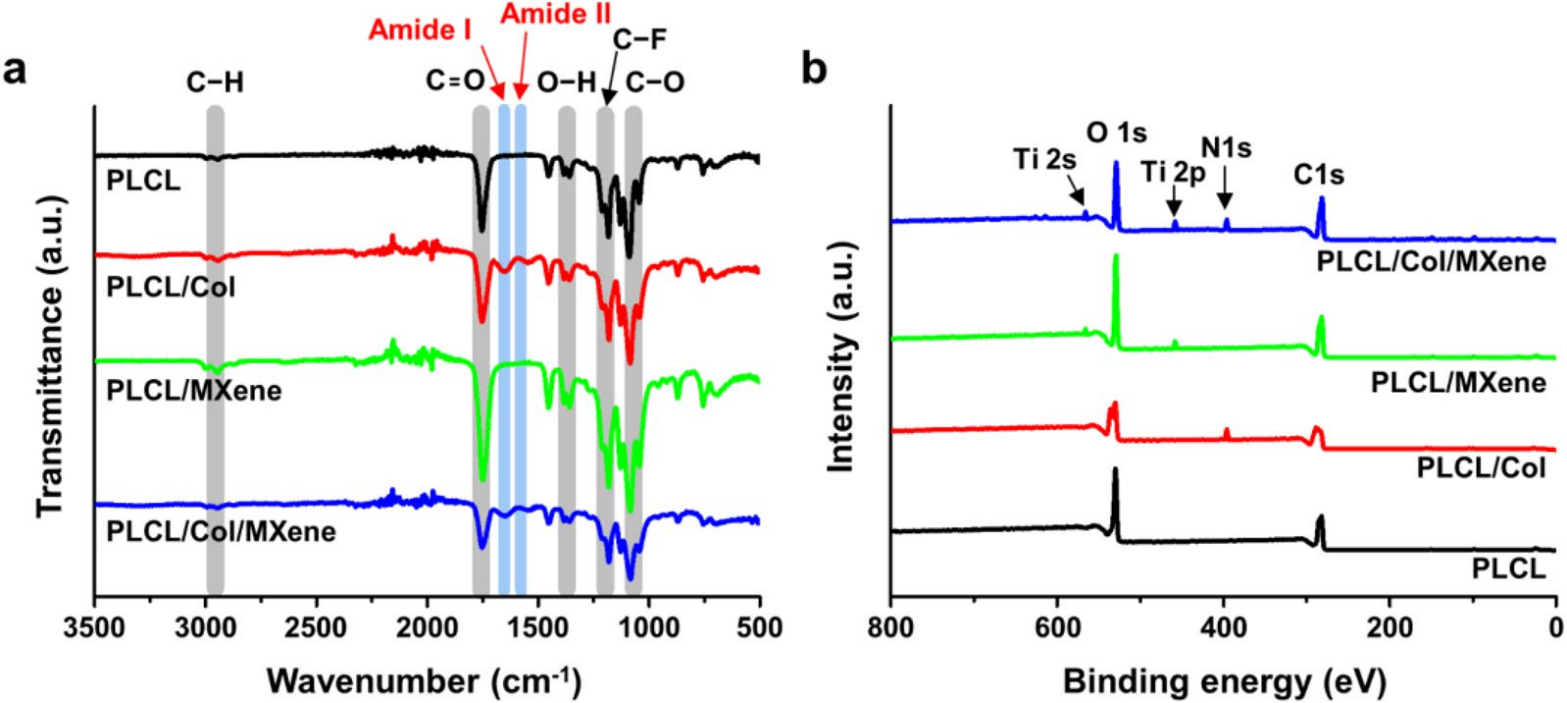
Chemical characterizations of PLCL, PLCL/Col, PLCL/MXene, and PLCL/Col/MXene nanofibrous matrices. **a** FT-IR spectra and **b** XPS spectra. Representative peaks are denoted with columns and arrows

To determine the chemical composition of each nanofiber, we used Fourier-transform infrared spectroscopy (FT-IR) spectroscopy and XPS as summarized in Fig. 3. In Fig. 3a, the bands of C−H, C=O, O−H, and C−O groups corresponding to PLCL were observed at 2990, 1754, 1384, and 1182 cm^‒1^ in the FT-IR spectrum of PLCL/Col/MXene nanofibers (labeled with gray column) [56, 57]. In addition, amide I and amide II bands from the Col were also observed near 1657 and 1552 cm^‒1^ (labeled with blue column) [58]. Moreover, additional bands at 1084, 1178, and 1452 cm^‒1^ (labeled with gray column) were assigned to C–O, C–F, and O–H bands, respectively, associated with MXene NPs. [59, 60]. As shown in Fig. 3b, the XPS spectra represented the characteristic peaks of PLCL, Col, and MXene NPs. In the pristine PLCL nanofibers, only the carbon and oxygen elements of the PLCL chain were denoted in C 1 s (~ 286 eV) and O 1 s peak (~ 530 eV), while N 1 s peak (~ 400 eV) was not observed [60]. Further, N 1 s peak (~ 395 eV) derived from the Col molecule was clearly displayed in the PLCL nanofibers in the hybridized structure [61, 62]. In the MXene NPs-decorated PLCL nanofibers, the two representative peaks of Ti from MXene NPs appeared as Ti 2p (~ 458 eV) and Ti 2 s (~ 566 eV), presenting Ti − O and Ti − C bond [63, 64]. The above set of results substantiated that MXene NPs with Col were successfully incorporated in the structure of PLCL/Col/MXene nanofibers.

### Cytotoxicity of MXene nanoparticles (NPs)

**Fig. 4:**
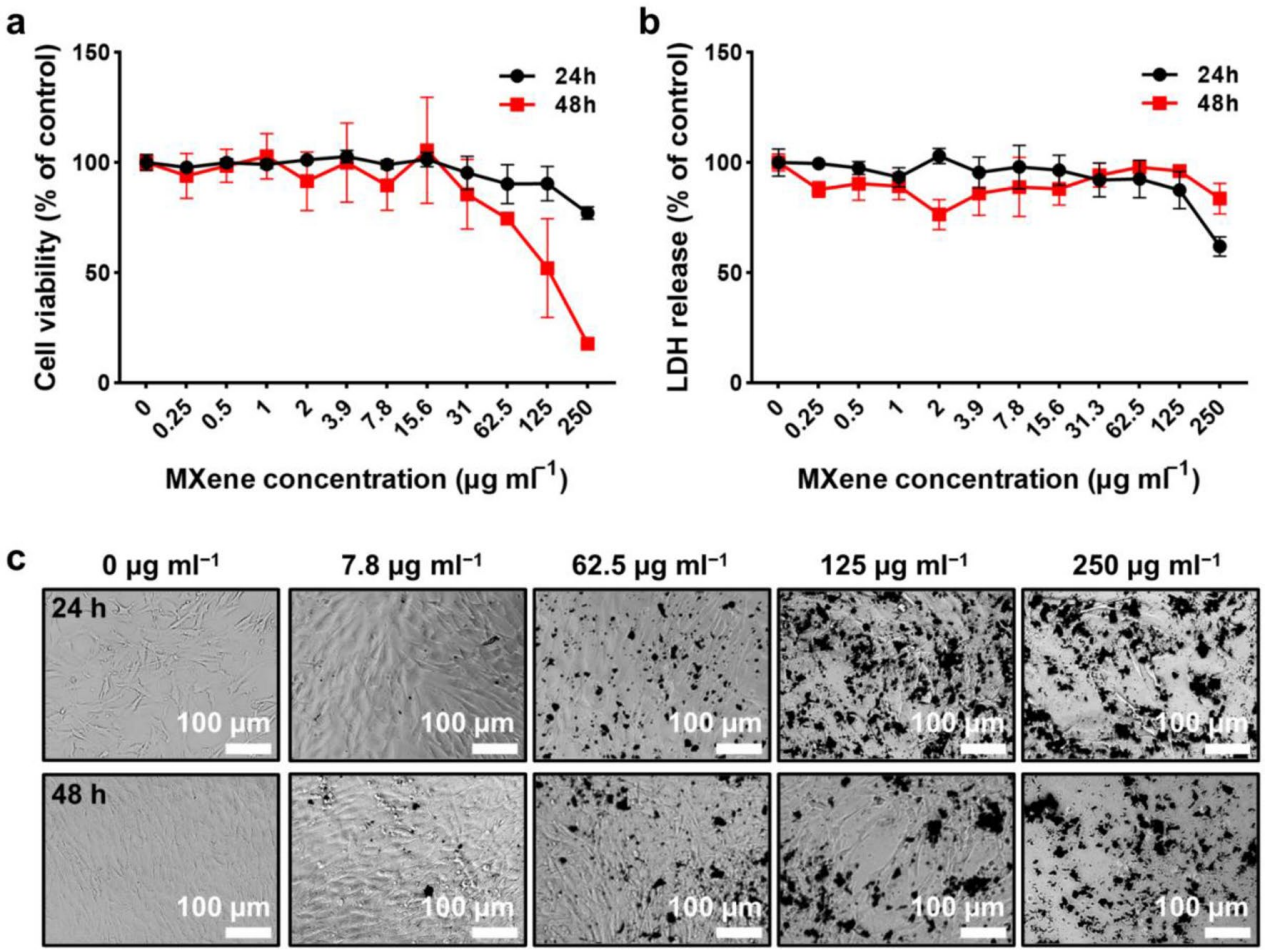
Cytotoxicity profiles of MXene NPs against MC3T3-E1 preosteoblasts. **a** CCK-8 assay and **b** LDH assay on MC3T3-E1 preosteoblasts treated with different concentrations of MXene NPs. **c** Morphological changes of MXene NPs-treated cells observed by optical microscopy. The data are expressed as the mean ± SD (n = 6). An asterisk (*) denotes a significant difference compared to the non-treated control, p < 0.05. All micrographs are representative of six independent experiments with similar results

The concentration range of MXene NPs can be an important parameter for a standard model system in nanofibrous polymeric scaffolds since the toxic inhaled events may inhibit potential role on the cultured cells. Thus, at the initial stage of the cell culture, the cytotoxicity of MXene NPs on MC3T3-E1 preosteoblasts was carefully evaluated on the cell metabolic activity and membrane integrity by typical cell counting kit-8 (CCK-8) and lactate dehydrogenase (LDH) assays, as presented in Fig. 4. First, the result from the CCK-8 assay show that the cell viability tends to decrease with increasing each splited concentration range up to 250 µg ml^‒1^ after 24 h and 48 h incubation (Fig. 4a). In particular, a correlation of the significant cytotoxicity (viability ≤ 80%) and specific concentration range were surveyed at 250 µg ml^‒1^ after 24 h and 62.5–250 µg ml^‒1^ after 48 h, which clarified that MXene NPs affected the activity of cells as a function of time, depending on the dose. As recently reported [65], the concentration range of MXene was surveyed by using in vivo model (i.e., zebrafish embryo model), in which a minimum dose-range was proposed for the biocompatible conditions that guides the dose-dependent uptake without significant teratogenic effects within the range of 100 mg ml^‒1^. Thus, the selected concentration range was reasonable in our experimental scheme. In addition to this result, as shown in Fig. 4b, a specific level of intracellular LDH release was investigated at the high concentration (i.e., 250 µg ml^‒1^ after 24 h). In general, LDH release tends to increase as cell membrane damage increases. However, our results showed a constant level of LDH tests on MXene NPs treatment in the range of 0–250 µg ml^‒1^ in spite of the high cytotoxicity. Thus, on the basis of the LDH test, it was confirmed that the integrity of the cell membrane was not disrupted by the loading of MXene NPs. Moreover, interestingly, the dead cells maintained their rounding shape rather than leaving a broken membrane, as presented in Additional file 1: Fig. S3. On the other hand, as presented in the graph and Fig. 4c, serious membrane damage with noticeable LDH release was detected after 48 h at the same concentration range. Because LDH is one of the sugar degradation-related enzymes in cells of various tissues, the extracellular LDH concentration rises when the cell membranes lose integrity to external damages [66–68]. By the fact that MXene NPs do not internalize into the cells without inducing stress, the only local transformation of the cytoskeletal structure can be derived from morphological changes, which leads to final detachment from the membrane surface. Based on this experimental background, the cell shape was transformed into a rounding shape, indicating a lower proliferation rate in the inactive state (Fig. 4c) [69]. Similar work also demonstrated that MXene material (i.e., Ti_3_C_2_) is cytocompatible to various types of cells, including A549, MRC-5, A-375, and HaCaT [70]. Because cytotoxicity can be closely related to the generation of oxidative stress by the oxidative stress and the related reactive oxygen species (ROS), a potential mechanism of toxicity is suggested with a slight impact on normal cells viability; thus, the concentration ranges are limited to a certain level (i.e., ~ 62.5 µg ml^‒1^). On this, a different class of MXene NPs exhibited little cytotoxicity at the concentration of 50 µg ml^‒1^, regardless of size, chemical composition, and functional group [68]. Mainly due to the bioinert properties of existing transition metal (e.g., Ti) and biocompatible C content, the cell viability in our approach was also maintained at the compatible range of ~ 62.5 µg ml^‒1^ concentration of MXene NPs [71]. Furthermore, compared to other types of 2D nanomaterials such as graphene derivatives, black phosphorus, and transition metal dichalcogenides, slightly higher levels of surface smoothness in the structured MXene NPs may derive less toxic responses of a lower level of membrane damage on the cultured cells [37, 72–74].

### Effects of MXene NPs on proliferation and ALP activity of MC3T3-E1 preosteoblasts

**Fig. 5:**
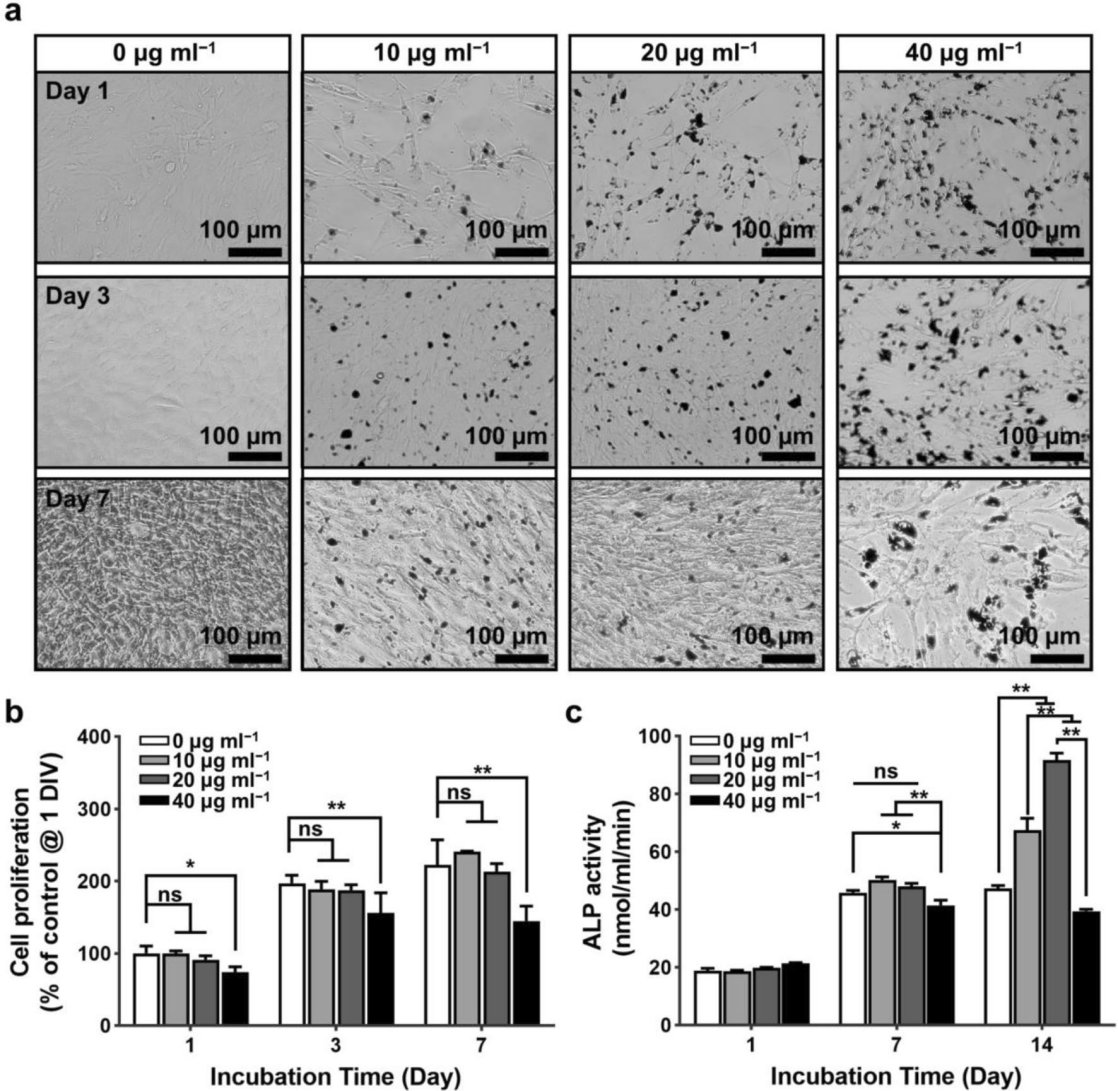
Effects of MXene NPs on proliferation and ALP activity of MC3T3-E1 preosteoblasts. **a** Morphological changes and **b** cell proliferation for 7 days, and **c** ALP activity for 14 days. All micrographs are representative of six independent experiments with similar results. The data are expressed as the mean ± SD (n = 6). Asterisks (* and **) denote a significant difference compared to the control, *p < 0.05 and **p < 0.01, while ‘ns’ denotes not significant

The growth and proliferation of preosteoblasts on the bone tissue scaffold play important role in osseointegration with natural tissue. As proven to be a ranged concentration of MXene in cytocompatibility (i.e., < 60 µg ml^‒1^) in the prior experimental result on the Mxene-added model, the morphology and proliferation of MC3T3-E1 preosteoblasts were evaluated by optical microscopy and a CCK-8 assay up to day 7 culture, as presented in Fig. 5a, b and Additional file 1: Fig. S4a. On the first day of incubation, no difference in cell proliferation was detected on the MXene NPs concentration range of 0–20 µg ml^‒1^, whereas the level of 40 µg ml^‒1^ showed significantly decreased cell proliferation (p < 0.05). In the following culture at 3–7 days, the cells treated with 10 and 20 µg ml^‒1^ of MXene NPs indicated a comparable proliferation rate with the control group (i.e., non-treated as grown). In contrast, a significantly decreased proliferation (p < 0.01) was observed at the cell treated with a more increased concentration of 40 µg ml^‒1^. At this stage, our results guided that the concentration range of the MXene NPs delicately mediated in long-term incubation, and the stable proliferation rate of preosteoblasts could be continued at the lower range of MXene content (i.e., below 40 µg ml^‒1^). This set of results also implies that the critical interactions with the cultured cells under a specific microenvironment will be an important parameter to control the cell behaviors and additional external stimuli. Therefore, to confirm the osteogenic activity of MXene NPs on MC3T3-E1 preosteoblasts, we tested ALP activity on each cultured cell as presented in Fig. 5c and Additional file 1: Fig. S4b. Interestingly, until to 7 days of culture, the cells treated up to 20 µg ml^‒1^ of MXene NPs did not show any significant difference in ALP activity, whereas the increased concentration range of 40 µg ml^‒1^ started to induce significantly decreased ALP activity (p < 0.01), compared to the other groups. More long-term periodic incubation (i.e., day 14), the cells treated with 10 and 20 µg ml^‒1^ of MXene NPs indicated remarkably increased ALP activity (p < 0.01) among other groups. However, in the case of the cells treated with MXene NPs of 40 µg ml^‒1^, the ALP activity showed a significantly decreased level (p < 0.01), a similar range to the level of 7 days of culture. This collective set of results is a good guidance in the selection of the concentration range to contrast the cell activities in the well-matched trend on the cell proliferation and the ALP activity. The most optimal concentration to host tissue cells was found to be ~ 20 µg ml^‒1^ of MXene NPs, compared to other groups by considering trade-off parametric conditions. Based on the previous seminal studies on the osteogenic activity of MXene NPs [12, 13, 35], MXene-decorated biocompatible scaffolds were designed to enhance the cell behaviors with optimized cellular transport kinetics.

### Cell behaviors of MC3T3-E1 preosteoblasts on PLCL/Col/MXene nanofibrous matrices

**Fig. 6:**
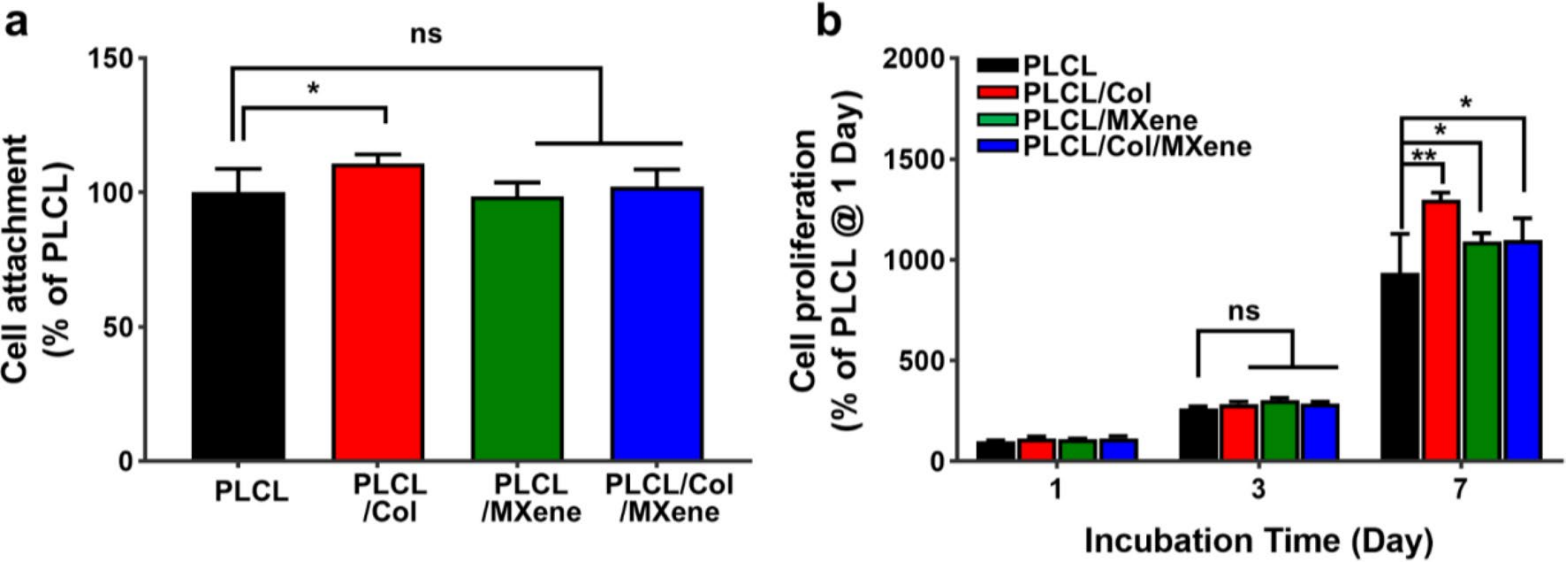
Attachment and proliferation of MC3T3-E1 preosteoblasts on PLCL, PLCL/Col, PLCL/MXene, PLCL/Col/MXene nanofibrous matrices. **a** Cell attachment at 6 h after seeding and **b** proliferation for 7 days of incubation on each matrix. The data are expressed as the mean ± SD (n = 6). Asterisks (* and **) denote a significant difference compared to the control, *p < 0.05 and **p < 0.01, while ‘ns’ denotes not significant

**Fig. 7:**
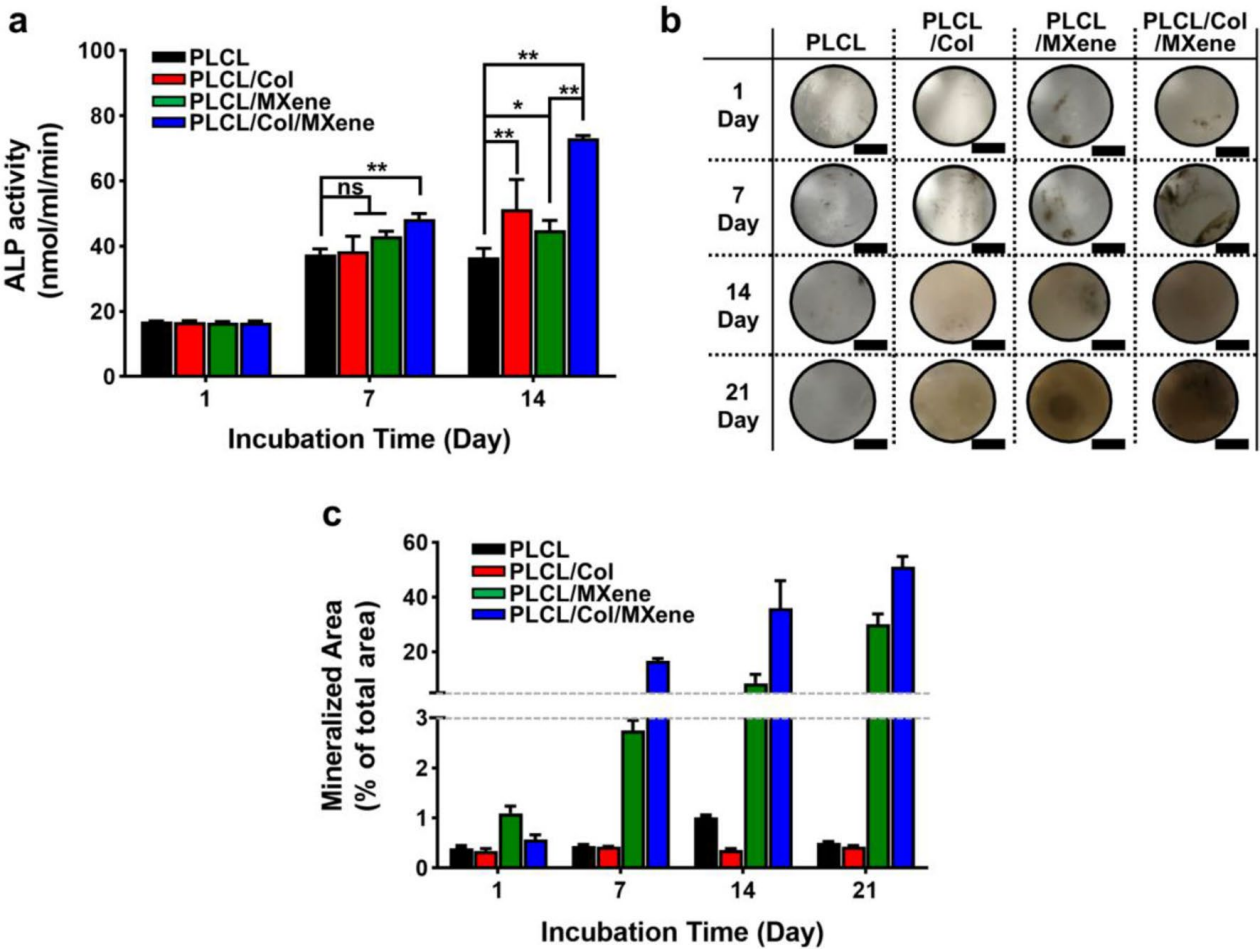
ALP activity and mineralized nodule formation of MC3T3-E1 preosteoblasts on PLCL, PLCL/Col, PLCL/MXene, PLCL/Col/MXene nanofibrous matrices. **a** ALP activity, **b** digital photographs of von Kossa-stained samples and **c** their colorimetric quantification. Quantitative analysis was performed using ImageJ software based on digital photographs of **b**. The mineralized bone nodules are stained with dark-brown color (scale bar = 5 mm). The data are expressed as the mean ± SD (n = 6). Asterisk (* and **) denote a significant difference compared to the control, *p < 0.05, while ‘ns’ denotes not significant. All photographs are representative of six independent experiments with similar results

The cell attachment and proliferation of MC3T3-E1 preosteoblasts on PLCL, PLCL/Col, PLCL/MXene, and PLCL/Col/MXene nanofibrous matrices were assessed by a CCK-8 assay. The initial attachment of preosteoblasts was significantly increased (p < 0.05) on the PLCL/Col nanofibrous matrices compared to the PLCL samples, in contrast to no significant difference between PLCL, PLCL/MXene, and PLCL/Col/MXene nanofibrous matrices (Fig. 6a). These results could be directly compared to another control group on the cultured cells on the generally used TCP, which represents that the initial cell adhesion on the fabricated nanofibrous matrices was in the range of ~ 60–70% (Additional file 1: Fig. S5). The proliferation of preosteoblasts was consistently increased during the culture period in all nanofibrous matrices (Fig. 6b). At 7 days of incubation, PLCL/Col and PLCL/Col/MXene nanofibrous matrices showed significantly increased cell proliferation (p < 0.01 and p < 0.05, respectively), compared to the PLCL nanofibrous matrices. These results suggest that increased initial attachment and hydrophilicity contribute to synergistically improving cell proliferation on the Col-hybridized PLCL nanofibrous matrix. As well known, previous studies have demonstrated that Col has excellent cytocompatibility and cell affinity to enhance cellular behaviors, such as initial attachment and proliferation [75, 76]. The featured cell adhesion can be one of the most important parameters in the preparation of optimized BTE scaffolds because the integrin-mediated cell attachment is highly favorable in bone cell growth and differentiation [77]. Moreover, recent studies have reported that more hydrophilic nanofibrous matrices in a certain range obviously facilitate several cellular behaviors [78–80]. The addition of Col in the matrix increases the hydrophilicity to regulate the expression of integrin subunit α_v_β_3_, and the type I Col itself also provides a specific cell-binding site to two members of the integrin subgroup, such as α_1_β_1_ and α_2_β_1_ integrins [77, 81] Because the initial cell adhesion was assessed after the short time condition of seeding (i.e., 6 h), the hydrophilic surface nature of the provided matrix was presumably the dominant factor rather than the biochemical reaction from the MXene NPs, so thus which resulted in only PLCL/Col matrix increased initial cell adhesion [82, 83]. MXene-based materials are known to promote cell–matrix interaction by supporting focal adhesion formation with filopodia protrusion and activating integrin-mediated signal pathways [12, 84]. In our experimental condition, this can be demonstrated by the result of increased cell proliferation rate on the PLCL/MXene and PLCL/Col/MXene, compared to other matrices. Hence, the lower level of cell proliferation in the PLCL/Col/MXene matrix represented an initiation of osteogenic differentiation by interrupting the proliferation rate (Fig. 7a). In the case of the PLCL and PLCL/MXene matrices, this effect was found to be less effective as a result of the relatively lower cell population, compared to other matrices. Although this simple method of improving cell affinity is an extension of the existing protocol, it was confirmed that an approach of a hybrid combination of polymeric matrices, such as PLCL/Col/MXene nanofibrous structure, played a rather important role based on our experimental results.

Finally, the osteogenic differentiation of MC3T3-E1 preosteoblasts was evaluated on the MXene-decorated nanofibrous matrices based on the analysis of ALP activity. Since the ALP activity is an early marker of osteogenic differentiation, noticeable trend identification can be important to determine osteogenic differentiation in specific microenvironments [85]. Figure 7a represented the ALP activity of preosteoblasts on each nanofibrous matrix until day 7, in which there were no marked variations in ALP activity for all matrices, except the cells cultured on the PLCL/Col/MXene matrices that showed significantly increased ALP activity (p < 0.01). After the incubation for up to 14 days, a clear difference was detected by the effective incorporation of Col and MXene NPs. Notably, the cells on PLCL/Col/MXene matrix significantly enhanced cellular behavior (p < 0.05) with increased ALP activity, compared to other control groups. However, interestingly, the cells on the PLCL/Col matrix showed a higher range of the ALP activity (p < 0.01) than the PLCL/MXene combination, representing an important role of the Col as observed in Fig. 6b. Due to the subtle combinatorial effects of MXene NPs and Col, ALP activity of cells on the PLCL/Col/MXene nanofibrous matrices indicated unexpected maximum value by the dual effect on osteogenic differentiation and encouraging cell stimulus. This result implies that the PLCL/Col/MXene nanofibrous matrices can promote the early osteogenic differentiation of preosteoblasts without the use of osteogenic agents. In addition, Von Kossa staining was used to monitor the biological mineralization of differentiated osteoblasts and to target carbonate or phosphate deposits [36, 40]. The late osteogenic differentiation of preosteoblasts was confirmed by assessing the formation of mineralized bone nodules to substantiate the biological role in the differentiated osteoblasts. As summarized in Fig. 7b, the digital images of the von Kossa-stained cells on the nanofibrous matrices clearly show a set of color maps. After the 14 days incubation of cells on the nanofibrous matrices, the mineralized bone nodules were stained in a dark-brown color only observed at the MXene-decorated scaffolds, while no apparent color changes were detected on the PLCL and PLCL/Col samples. Moreover, when the mineralized area of bone nodules was quantitatively extracted from the images by ImageJ software analysis, the distribution of pixels identified the effective bone mineralization in the provided microenvironments as appeared in Fig. 7c. At 14 and 21 days, the MXene-decorated matrices remarkably increased mineralized area (p < 0.05), directly compared to the MXene-free scaffolds. These results demonstrated that MXene NPs exerted stronger attractive cell interactions to accelerate the late-stage osteogenic differentiation with no need for osteogenic factors. This interesting phenomenon may be attributed to the accelerated adsorption of Ca^2+^ ions, originating from the intrinsic cytocompatibility of the MXene NPs, as previously speculated based on first-principles theory [86]. As presented in Fig. 1g, the partially oxidized surface of the MXene NPs illustrated the valuable features of the chemical composition of negatively charged TiO_2_ and corresponding remarkable cytocompatibility due to the high binding affinity of the Ca^2+^ ions adsorption on the provided surface. Instead of freshly prepared MXene solution, we aged MXene NPs in DI water for 3 weeks after the synthesis. This finding delivers somewhat interesting information on the osteoblastic environment and implies that a new approach to osteogenic differentiation can be proposed in a view of the ideal condition for maximizing the beneficial cellular behavior induced by MXene NPs.

Therefore, we postulate that the osteogenic effects of MXene NPs as a more attractive potent cue can be attributed to the following reasons. The osteogenic differentiation of MC3T3-E1 preosteoblasts on MXene-loaded matrices could be influenced by both topographical features of the surface morphology and appreciated elements from the secondary phase of the MXene, exhibiting improved cytocompatibility comparable to the pure Ti. It also should be acknowledged that the functional groups of MXene NPs introduce negative charges creating an electrically charged microenvironment suitable for bone regeneration, based on recent studies [87, 88]. Moreover, the hydroxy groups of MXene are known to form hydrogen bonds with serum proteins that support the synergistic behavior of the preosteoblasts in terms of initial adhesion and proliferation [46]. Meanwhile, the carbon element containing MXene might have the ability to promote osteogenic differentiation, similar to other carbon-based nanomaterials, possessing osteogenic effects [89–91]. In addition, novel bionanomaterials similar to ECM-mimicking nanotopography that can influence the intrinsic behavior of MC3T3-E1 cells have been used to promote osteogenic differentiation. Wang et al*.* introduced that the M13 phage nanofiber matrices promoted osteogenic differentiation of induced pluripotent stem cells (iPSCs) by causing the elongation of cells following the ridge/groove nanotopographical cues with the presence of adhesive and osteogenic signaling peptides [92]. Another study on chitosan/gelatin films confirmed that nanotopography activation pathways, such as integrin-mediated signaling cascades and local adhesion, induced transcriptional regulatory effects by upregulating osteogenic differentiation-associated RUNX2, OCN, and OPN genes [93]. Furthermore, Furthermore, the high affinity of MXene for ions can be inferred as one of the dominant factors in osteogenesis facilitation. Maureira et al*.* fabricated bioactive glass nanoparticle-loaded chitosan–gelatin hydrogel beads [94]. The phosphate and calcium component of bioactive glasses could be converted to hydroxyapatite to stimulate osteogenic differentiation through different pathways [95]. The ions from the bioactive glass in a nanocomposite form activated the osteoblast genotype expression of dental pulp stem cells by upregulation of ALP activity in vitro. They also demonstrated excellent bone tissue formation after 8 weeks of implantation in vivo [94]. Consequently, similar to the other reported materials systems, the PLCL/Col/MXene nanofibrous matrices could provide an excellent microenvironment with enhanced cell adhesion, growth, and proliferation, which suggest a wide range of potential BTE scaffolds that can effectively drive spontaneous osteogenesis with no use of osteogenic factor applications.

## Conclusions

In summary, we developed a ternary nanofibrous MXene NPs-integrated PLCL/Col scaffold, produced simply by electrospinning process to exquisitely evaluate bone tissue regeneration. The outcomes indicated that the PLCL/Col/MXene nanofibrous matrix exhibited structural similarity with natural ECM and excellent physicochemical properties, which can provide favorable microenvironments for unprecedented cellular behaviors of MC3T3-E1 preosteoblasts. As designed in our scheme, the incorporation of Col-mediated PLCL nanofibrous matrices enhanced the initial cell adhesion and proliferation, while the MXene NPs-decorated matrices promoted cell proliferation to some extent. Furthermore, those MXene NPs-decorated matrices exhibited exceptional osteogenic activity that led to explicitly spontaneous osteogenic differentiation of preosteoblasts. As an on-demand concept, our strategy on the potential use of the PLCL/Col/MXene nanofibrous scaffold is of importance by directing cellular responses in the context of the development of biofunctional 2D nanomaterials to promote bone regeneration. Taken together, our observations on the newly combined biomaterial suggest that the Mxene-mediated nanostructured arrays can be a promising candidate, extending to other types of cells or tissues that provide an amicable matrix to find critical signaling pathways toward cellular behaviors as well as accelerating osteogenesis.

## Supplementary Information

**Additional file 1: Table S1. **Electrospinning condition of each nanofibrous matrices. **Fig. S1.** TEM image of layered Ti_3_C_2_T_x_ and nanosheet. **Fig. S2.** SEM image of layered Ti_3_C_2_T_x_. **Fig. S3. **Optical micrographs of LDH assay at the concentration of 250 μg ml^-1^ of MXene NPs. The round shape of the dead cells were marked in yellow circles. After 24 h of incubation (a), and after 48 h of incubation (b). (c–d) The highly magnified images from the yellow-marked area shown in (a–b). **Fig. S4. **Effects of MXene NPs on proliferation and ALP activity of MC3T3-E1 preosteoblasts. (A) Cell proliferation for 7 days, and (B) ALP activity for 14 days. All micrographs are representative of six independent experiments with similar results. The data are expressed as the mean ± SD (n = 6). Asterisks (* and **) denote a significant difference compared to the control, * p <0.05 and ** p <0.01, while ‘ns’ denotes not significant. **Fig. S5. **Attachment of MC3T3-E1 preosteoblasts on tissue culture plastic (TCP), PLCL, PLCL/Col, PLCL/MXene, and PLCL/Col/MXene nanofibrous matrices. Attachment was measured using a CCK-8 assay at 6 hours after seeding. The data are expressed as the mean ± SD (n = 6). Asterisks (* and **) denote a significant difference compared to the control, * p <0.05 and ** p <0.01, while ‘ns’ denotes not significant.

## Data Availability

The datasets used and/or analyzed during the current study are available from the corresponding author on reasonable request.
