# Supplementary material for: Ternary MXene-loaded PLCL/collagen nanofibrous scaffolds that promote spontaneous osteogenic differentiation
Source: Nano Converg. 2022 Aug 27;9:38. doi: 10.1186/s40580-022-00329-3 (PMC9420163; doi:10.1186/s40580-022-00329-3)
Supplement: Supplementary file 1 — Additional file 1: Table S1. Electrospinning condition of each nanofibrous matrices. Fig. S1. TEM image of layered Ti3C2Tx and nanosheet. Fig. S2. SEM image of layered Ti3C2Tx. Fig. S3. Optical micrographs of LDH assay at the concentration of 250 μg ml-1 of MXene NPs. The round shape of the dead cells were marked in yellow circles. After 24 h of incubation (a), and after 48 h of incubation (b). (c–d) The highly magnified images from the yellow-marked area shown in (a–b). Fig. S4. Effects of MXene NPs on proliferation and ALP activity of MC3T3-E1 preosteoblasts. (A) Cell proliferation for 7 days, and (B) ALP activity for 14 days. All micrographs are representative of six independent experiments with similar results. The data are expressed as the mean ± SD (n = 6). Asterisks (* and **) denote a significant difference compared to the control, * p <0.05 and ** p <0.01, while ‘ns’ denotes not significant. Fig. S5. Attachment of MC3T3-E1 preosteoblasts on tissue culture plastic (TCP), PLCL, PLCL/Col, PLCL/MXene, and PLCL/Col/MXene nanofibrous matrices. Attachment was measured using a CCK-8 assay at 6 hours after seeding. The data are expressed as the mean ± SD (n = 6). Asterisks (* and **) denote a significant difference compared to the control, * p <0.05 and ** p <0.01, while ‘ns’ denotes not significant. [file 40580_2022_329_MOESM1_ESM.docx]

**Additional file 1**

Ternary MXene-loaded PLCL/collagen nanofibrous scaffolds that promote spontaneous osteogenic differentiation

Seok Hyun Lee^1,†^, Sangheon Jeon^1,†^, Xiaoxiao Qu^1^, Moon Sung Kang^1^, Jong Ho Lee^2^, Dong-Wook Han^1,3,*^, Suck Won Hong^1,4,*^

^1^Department of Cogno-Mechatronics Engineering, College of Nanoscience and Nanotechnology, Pusan National University, Busan 46241, Republic of Korea

^2^ Daan Korea Corporation, Seoul 06252, Republic of Korea

^3^ BIO-IT Fusion Technology Research Institute, Pusan National University, Busan 46241, Republic of Korea

^4^ Engineering Research Center for Color-Modulated Extra-Sensory Perception Technology, Pusan National University, Busan 46241, Republic of Korea

^†^These authors contributed equally to this work.

**Table S1.** Electrospinning condition of each nanofibrous matrices.

| **Experimental group** | **Composition** | **Needle**  **(mm)** | **Distance**  **(cm)** | **Flow rate**  **(ml h^-1^)** | **Voltage**  **(kV)** | **Solvent** |
| --- | --- | --- | --- | --- | --- | --- |
| **PLCL** | **5 %(w/v)** | **0.51** | **9** | **0.2** | **16** | **HFIP** |
| **PLCL/Col** | **5/0.5 %(w/v)** |  |  |  |  |  |
| **PLCL/MXene** | **5 %(w/v) + 400 µg ml^-1^** |  |  |  |  |  |
| **PLCL/Col/**  **MXene** | **5/0.5 %(w/v) + 400 µg ml^-1^** |  |  |  |  |  |

**
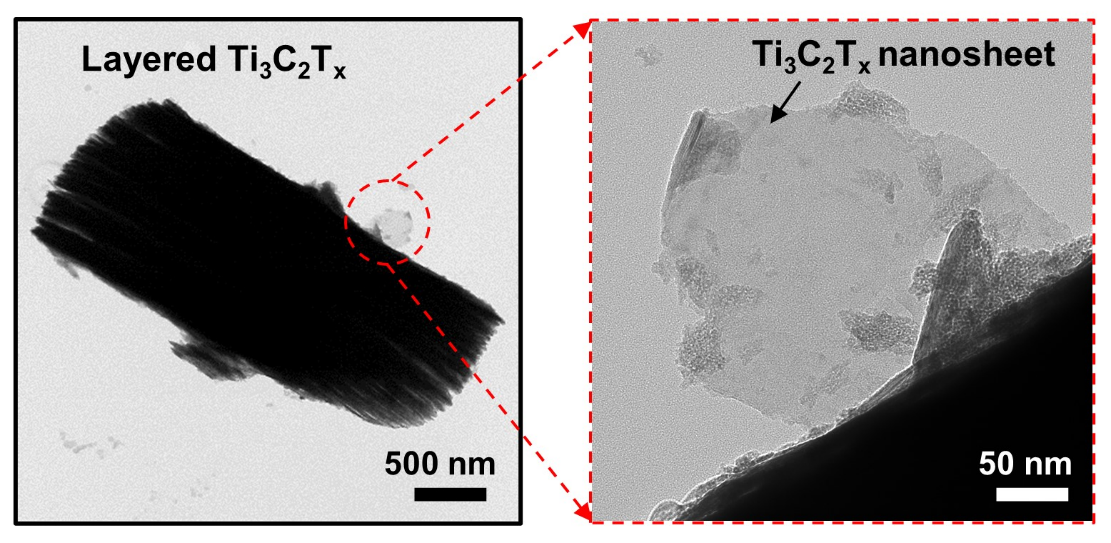
**

**Fig. S1.** TEM image of layered Ti_3_C_2_T_x_ and nanosheet.

**
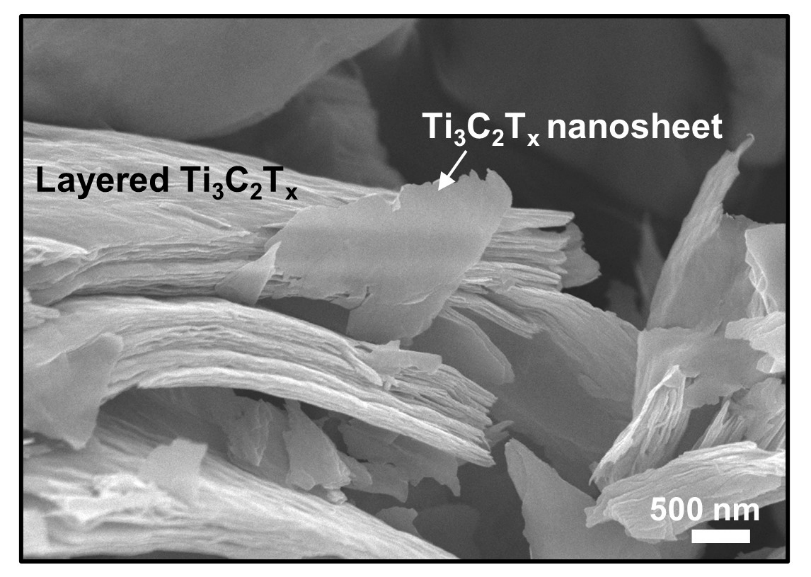
**

**Fig. S2.** SEM image of layered Ti_3_C_2_T_x_.

**
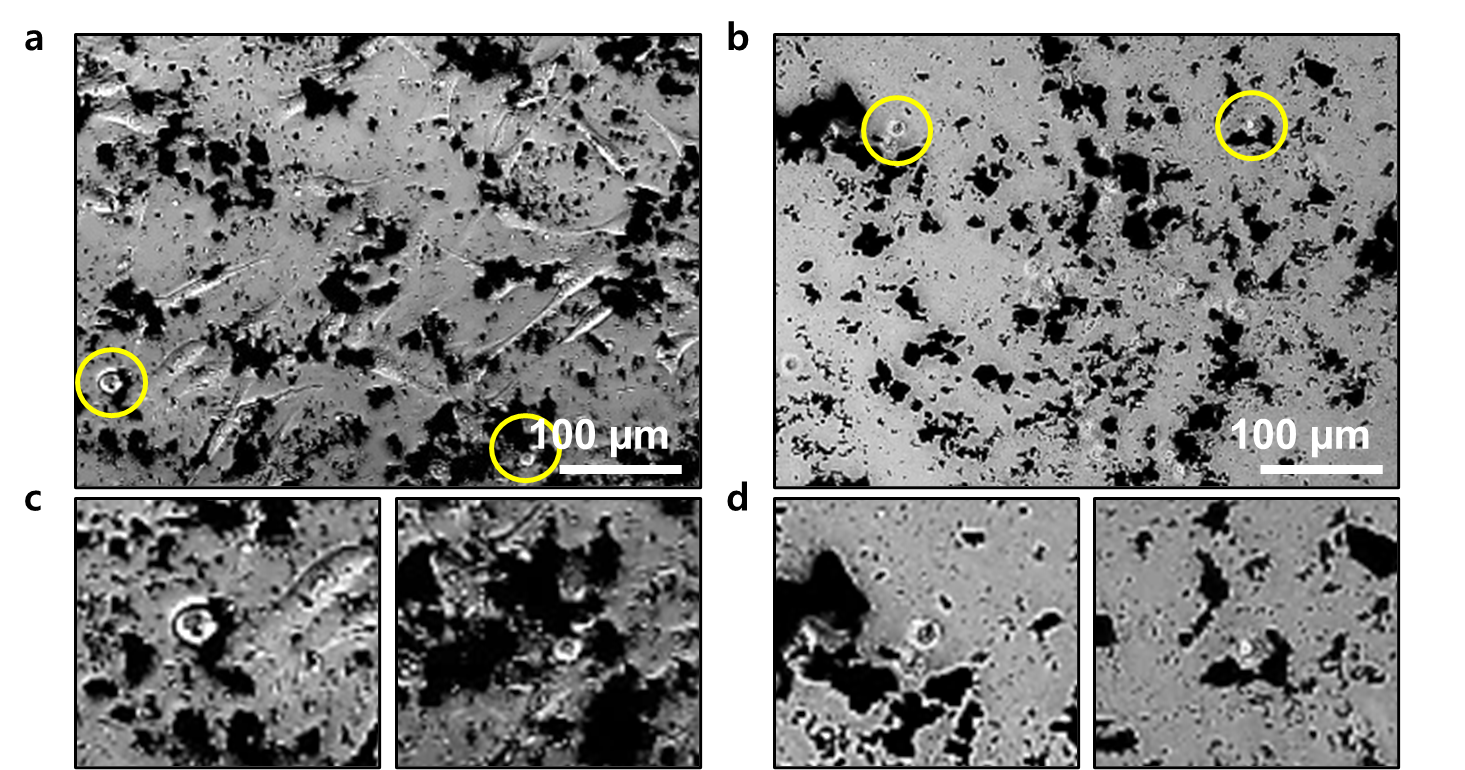
**

**Fig. S3. Optical micrographs of LDH assay at the concentration of 250 μg ml^-1^ of MXene NPs.** The round shape of the dead cells were marked in yellow circles. After 24 h of incubation (a), and after 48 h of incubation (b). (c-d) The highly magnified images from the yellow-marked area shown in (a-b).

**
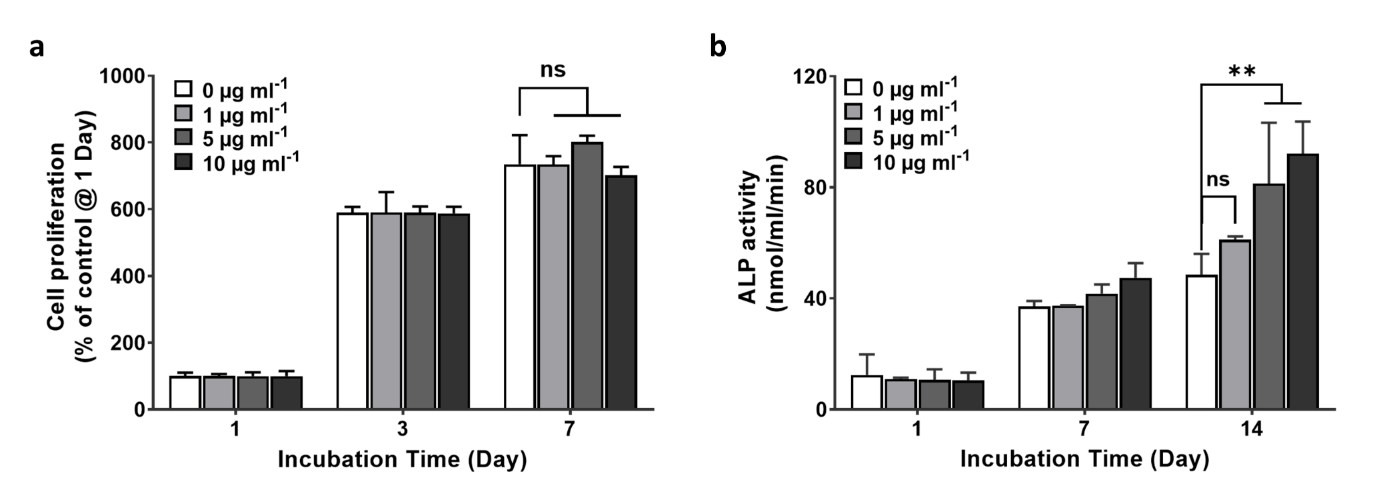
**

**Fig. S4.** Effects of MXene NPs on proliferation and ALP activity of MC3T3-E1 preosteoblasts. (A) Cell proliferation for 7 days, and (B) ALP activity for 14 days. All micrographs are representative of six independent experiments with similar results. The data are expressed as the mean ± SD (n = 6). Asterisks (* and **) denote a significant difference compared to the control, * p <0.05 and ** p <0.01, while ‘ns’ denotes not significant.

**
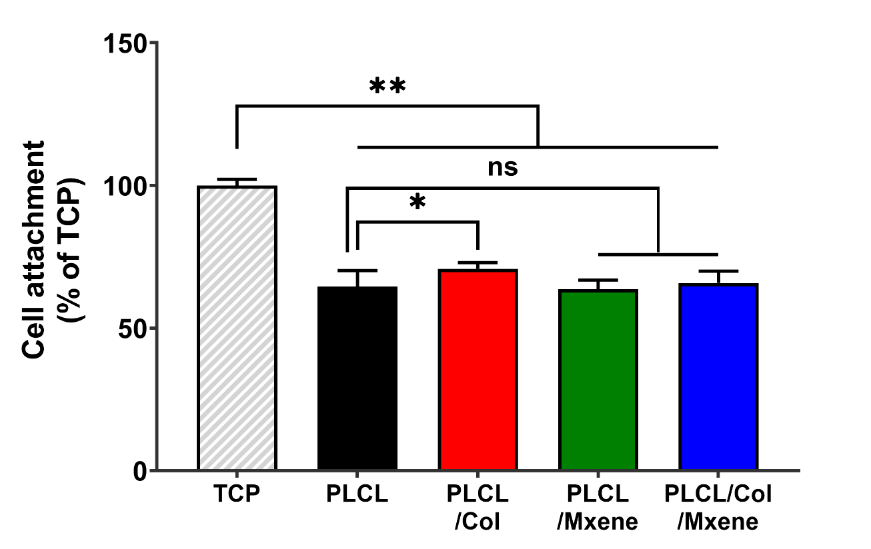
**

**Fig. S5.** Attachment of MC3T3-E1 preosteoblasts on tissue culture plastic (TCP), PLCL, PLCL/Col, PLCL/MXene, and PLCL/Col/MXene nanofibrous matrices. Attachment was measured using a CCK-8 assay at 6 hours after seeding. The data are expressed as the mean ± SD (n = 6). Asterisks (* and **) denote a significant difference compared to the control, * p <0.05 and ** p <0.01, while ‘ns’ denotes not significant.
